# Combination Therapy in Renal Cell Carcinoma: the Best Choice for Every Patient?

**DOI:** 10.1007/s11912-021-01140-9

**Published:** 2021-11-08

**Authors:** Ernesto Rossi, Melissa Bersanelli, Alain Jonathan Gelibter, Nicolò Borsellino, Claudia Caserta, Laura Doni, Marco Maruzzo, Alessandra Mosca, Carmela Pisano, Elena Verzoni, Paolo Andrea Zucali

**Affiliations:** 1 Medical Oncology, Fondazione Policlinico Universitario Agostino Gemelli IRCCS, Rome, Italy; 2 Medicine and Surgery Department, University of Parma and Medical Oncology Unit, University Hospital of Parma, Parma, Italy; 3 Medical Oncology Unit B, “Sapienza” University of Rome, Policlinico Umberto I, Rome, Italy; 4 Medical Oncology, Buccheri La Ferla – Fatebenefratelli Hospital, Palermo, Italy; 5 Medical and Translational Oncology, Azienda Ospedaliera Santa Maria, Terni, Italy; 6 Medical Oncology, Azienda Ospedaliero Universitaria Careggi, Firenze, Italy; 7 Medical Oncology Unit 1, Department of Oncology, Istituto Oncologico Veneto IOV IRCCS, Padova, Italy; 8 Multidisciplinary Outpatient Oncology Clinic, Candiolo Cancer Institute, FPO-IRCCS, Candiolo, Turin, Italy; 9 Department of Urology and Gynecology, Istituto Nazionale Tumori IRCCS Fondazione G. Pascale, Napoli, Italy; 10 Medical Oncology, Fondazione IRCCS Istituto Dei Tumori, Milan, Italy; 11 Department of Biomedical Sciences, Humanitas University, Pieve Emanuele, Milan, Italy; 12 Department of Oncology, IRCCS Humanitas Research Hospital, Rozzano, Milan, Italy

**Keywords:** mRCC, Combination therapy, Antiangiogenic factors, Tyrosine kinase inhibitors (TKI), Immune checkpoint inhibitors (ICI), Immunotherapy

## Abstract

**Purpose of Review:**

Therapeutic alternatives to treat metastatic renal cell carcinoma (mRCC) are increasing, and combination therapies, including antiangiogenic agents and tyrosine kinase/mTOR/immune checkpoint inhibitors, are identified as the gold standard driven by the results of recent clinical studies. Nevertheless, the real-world RCC population is very heterogeneous, with categories of patients not represented in the enrolled trial population who may not benefit more from these treatments. The purpose of this expert review is to assess the rationale on which tyrosine kinase alone may still be a viable first-line treatment option for some subgroups of patients with mRCC.

**Recent Findings:**

The first-line treatment with tyrosine kinase inhibitor monotherapy can still be considered an effective tool for addressing selected mRCCs, as highlighted by the successful outcome in a range of subjects such as favorable-risk patients, the ones suffering from autoimmune diseases, those with pancreatic or lung metastases, or previously undergoing organ transplantation and elderly subjects.

**Summary:**

Some selected categories of patients may still benefit from monotherapy with TKI, and smart sequential therapies can also be considered instead of a combination strategy. Tyrosine kinase inhibitors can also act as immune modulator agents, boosting the immune response to facilitate and potentiate the therapeutic effectiveness of subsequent immunotherapy.

## Introduction

The therapeutic armamentarium for the treatment of metastatic renal cell carcinoma (mRCC) is increasingly rich nowadays. The definition of a therapeutic algorithm is a crucial issue, considering the complexity of the factors involved in the progression of the disease and the heterogeneity of the population affected. Because of the lack of validated predictive factors, the choice of treatment is made even more difficult. Furthermore, the characteristics of “real life” patients may be different from those enrolled in clinical trials.

The current treatments for mRCC include systemic therapies such as several tyrosine kinase inhibitors (TKIs) or immune checkpoint inhibitors (ICIs) [1••, 2]. Such therapies can be administered sequentially, but, more recently, combination therapies have been introduced with the aim to improve patient outcomes. These therapies act synergistically on different targets and pathways involved in tumor progression. As suggested by the promising results of recent clinical trials, combination therapies are believed to be of greater importance in the treatment of RCC [3•, 4•, 5••, 6, 7•]. While combined therapies are becoming the standard of treatment, it is not entirely clear whether such approaches are the best option for all patients. Indeed, the purpose of this expert review is to assess the rationale on which TKI alone may still be a viable first-line treatment option for some subgroups of patients with mRCC.

## Monotherapy vs Combination Therapy

Tumor neo-angiogenesis is a key event in tumor progression, and several drugs with antiangiogenic activity as TKIs or bevacizumab have proven to be effective in first-line treatment [8]. In phase III studies, conducted mainly on good- or intermediate-risk patients, sunitinib showed a stark improvement in progression-free survival (PFS) compared to interferon alfa (IFN-α) [9, 10].

In addition, pazopanib has shown non-inferiority to sunitinib in the phase III COMPARZ study [11]. More recently, cabozantinib, a TKI which acts on various receptors such as hepatocyte growth factor receptor, vascular endothelial growth factor receptor (VEGFR), AXL, and FLT3, showing multiple inhibitory effects on angiogenesis, cell proliferation, migration and invasion, and solid tumor growth [12], also showed, as a primary endpoint, prolonged PFS (8.6 months, 95% CI 6.8–14.0 vs 5.3 months, 95% CI 3.0–8.2; hazard ratio [HR] 0.48, 95% CI 0.31–0.74). In this CABOSUN phase II trial, cabozantinib was used as first-line with respect to sunitinib on 157 intermediate and poor-risk patients [13]. Cabozantinib improved PFS – the primary endpoint of the study – (HR 0.51 [95% CI 0.41–0.66], *p* < 0.001), overall survival (OS) (21.4 vs 16.5 months, HR 0.66 [95% CI 0.50–0.85]), and objective response rate (ORR) (17% vs 3%, *p* < 0.001) (both secondary endpoints), in second- and third-line treatment, compared to everolimus. A favorable clinical outcome was established during the follow-up of the study, irrespective of the type of the first treatment, either VEGFR inhibitor or ICIs [14].

In recent clinical trials, the introduction of TKIs (or anti-VEGF agent) in combination with new ICIs that interfere with programmed cell death protein 1 (PD-1) [15] showed remarkable results overall.

The combination of bevacizumab with atezolizumab, a monoclonal antibody against the protein programmed cell death-ligand 1 (PD-L1), has been compared with sunitinib in the IMmotion151 study as a first-line treatment in mRCC PD-L1 positive patients. Primary endpoints were PFS in patients expressing PD-L1 and OS in the intention-to-treat population. In the study, the association bevacizumab + atezolizumab was found to be superior over sunitinib in terms of PFS (11.2 months vs 7.7 months, HR 0.74, *p* = 0.02) in PD-L1 positive patients, while no significant improvement in OS was reported [3•].

Moreover, the randomized, open-label, phase III trial JAVELIN Renal 101 demonstrated an advantage with the association of avelumab + axitinib vs sunitinib in previously untreated patients – PD-L1 positive, as shown by the primary endpoint result (PFS in PD-L1 patients 13.8 vs 7.2 months; HR 0.61; *p* < 0.001) and also by ORR in PD-L1 positive (a secondary endpoint, 55.2% vs 25.5%), regardless of patient risk category [4•]. A statistically significant advantage in PFS and ORR has also been reported in the general population. The results for the experimental combination in terms of OS in the PD-L1 population, another primary endpoint, are immature at the moment to be properly discussed [16].

Recently, the efficacy of the combination pembrolizumab–axitinib vs sunitinib was investigated in an open-label, phase III trial involving 861 patients with previously untreated advanced RCC. Primary endpoints were OS and PFS in the intention-to-treat population. The results for the combination showed a 47% reduction in death risk and 31% in disease progression compared to the monotherapy, respectively [17]. Nevertheless, the combination therapy did not show a significant benefit in the subgroup of favorable-risk patients (HR for OS: 0.64; 95% CI, 0.24–1.68) [17].

The CLEAR study, a multicenter open-label, randomized, phase III trial showed a significantly longer PFS (primary endpoint of the study, median 23.9 vs. 9.2 months; HR for disease progression or death, 0.39; 95% CI, 0.32 to 0.49; *p* < 0.001) for the combination lenvatinib–pembrolizumab vs sunitinib monotherapy; HR for death was 0.66 (95% CI, 0.49 to 0.88; *p* = 0.005). In the same study, significantly better PFS was found for the combination lenvatinib–everolimus (without significant OS difference) as first-line treatment of patients with advanced RCC [7•]. The results of this trial are outstanding and definitely introduce a new first-line option for advanced RCC patients, with possible hints on the lenvatinib’s potential immunomodulatory activity [7•]. In this case, the favorable-risk subgroup reached a significant PFS benefit (primary endpoint) but still did not reach significant benefit in terms of OS (secondary endpoint) (HR 1.15; 95% CI, 0.55–2.40).

The combination nivolumab–cabozantinib is also being tested in a phase III study, the CheckMate 9ER. The results of the study at median follow-up of 18.1 months showed superiority of the combination in terms of PFS (primary endpoint of the study,16.6 v 8.3 months, HR 0.51; 95% CI 0.41–0.64, *p* < 0.001), OS at 12 months was 85.7% vs 75.6% (secondary endpoint; HR 0.60 [98.89% CI 0.40–0.89]; *p* < 0.001), and ORR (secondary endpoint; 55.7% [50.1–61.2] vs 27.1% [22.4–32.3]; *p* < 0.001) compared to sunitinib, in first-line treatment of all the subgroups analyzed. In addition, 8.0% of the individuals in combination therapy achieved a complete response, compared to 4.6% in the sunitinib group. These results were also supported by a manageable safety profile [18].

A recent phase III study (CONTACT 03) comparing the combination of cabozantinib and atezolizumab vs cabozantinib monotherapy has already begun as a second-line treatment for patients treated with ICI [19]. This study will test the efficacy and safety (primary endpoints: PFS and OS; secondary endpoints: ORR and safety among others) of the combination vs monotherapy and investigate the efficacy of sequential treatments, in this case, a combination of a TKI and a PD-L1 inhibitor or a TKI alone after an ICI.

Another large phase III study (CHECKMATE 214), conducted on 1096 untreated mRCC patients, demonstrated an improvement in OS and ORR (both primary endpoints) with the combination of two ICIs, nivolumab, targeting PD-L1, and ipilimumab, a monoclonal antibody which inhibits cytotoxic T-lymphocyte-associated antigen 4 (CTLA-4) [20], compared to sunitinib, in intermediate- and poor-risk patients [5••]; these data were confirmed by the follow-up. Of note, the primary endpoint population was the intermediate–poor-risk patient group, the intention-to-treat population including all-risk patients. Following a median of > 32 months in favorable-risk patients, the PFS and OS were found to be similar between the two arms of treatment in this subgroup, with a greater proportion of patients achieving objective response with sunitinib (50% vs 39%, *p* = 0.14) [21]. Interestingly, the benefit of the combination treatment vs sunitinib was even more evident in intermediate/poor-risk patients with sarcomatoid carcinoma, likely due to the worse prognosis of sarcomatoid cases treated with TKI monotherapy as the comparator. In this sarcomatoid carcinoma subgroup, 56.7% vs 19.2% (*p* < 0.001) of the patients reached an ORR and 18.3% vs 0% patients showed a complete response with the combination therapy compared to the monotherapy, respectively. These data are sustained by a safe profile, and prospective studies are underway to better the efficacy assessment of nivolumab/ipilimumab in this subgroup of patients [22].

Recently, the phase III TiNivo-2 trial has been announced to test both the activity and the tolerability of the combination tivozanib–nivolumab vs tivozanib monotherapy [23]. Tivozanib is a TKI with a potent action specifically on VEGF-1, 2, 3 [24]. Tivozanib was shown to be effective as a third- or fourth-line treatment compared to sorafenib in phase III TIVO-3 trial [25]. The phase I/II trial showed that the combination tivozanib–nivolumab is comparable to other TKI/PD-1 combinations in terms of frequency and severity of adverse events [4•, 17, 26].

Furthermore, the combination belzutifan (a hypoxia-inducible factor 2α inhibitor)-cabozantinib is being evaluated in a phase II trial in advanced RCC patients either naïve or who have already received 1 or 2 previous treatments. Preliminary results for the arm of patients who were already treated indicate good tolerability, with no grade 4 TRAEs registered after 24 months (median time from enrollment to data cutoff 11.3 months, 5.6–24.0 months) [27•, 28].

The trials investigating the combination therapies show promising and exciting results. However, most of the patients enrolled in clinical studies are characterized by overall good performance status and the absence of relevant comorbidities. This condition is most likely unmet in the daily routine, where a quite heterogeneous population is expected to be treated [29]. It has been estimated that oncology accounts for < 5% of patients enrolled in clinical trials [30], and consequently, the study population does not entirely represent those observed during daily clinical practice.

Therefore, it is necessary to consider specific patient characteristics in order to define the best treatment, as a combination therapy may not always be the optimal choice, and other alternatives, such as the therapy with the TKI alone, may still be considered.

## Combination Therapy Versus TKI Alone in Selected Patient Subgroups

Combination therapy has been shown to be superior to monotherapy in clinical trials [3•, 5••, 17]. Nevertheless, there are subgroups of patients for whom the association of agents could not be the optimal choice, resulting not applicable due to the patient characteristics. This is the case for patients who suffer from autoimmune diseases. Additionally, there is a lack of clear evidence of the beneficial effects of combination therapy in the subgroup of favorable-risk patients, as shown by the subgroup analyses from pivotal clinical trials. Regarding the group of favorable-risk patients, the Checkmate 9ER trial reports the efficacy of the combination nivolumab plus cabozantinib in all the categories of patients (favorable, intermediate, and poor-risk), although the published data report that the follow-up is still ongoing and the study is not mature to assess the survival in the favorable-risk group [18].

In the KEYNOTE 426 study, the pembrolizumab–axitinib combination showed superiority over sunitinib in terms of OS, PFS, and ORR [17]. However, the follow-up of the study data presented at the ASCO Annual Meeting 2020 showed a lack of maintenance in good-risk patients in terms of PFS and OS, with no significant differences between both combination and monotherapy after 23 months [31].

In addition, the CHECKMATE 214 study with nivolumab/ipilimumab, which reported beneficial effects for most of the patients, failed to show any improvement in those with a favorable prognosis, showing a higher OS in the sunitinib group [5••]. Moreover, first-line cabozantinib monotherapy was effective specifically in poor–intermediate-risk patients, as evident in the phase II CABOSUN trial [13, 32].

A recent report compares first-line combination treatments (immune-modulation + anti-VEGF vs ipilimumab + nivolumab) within the intermediate/poor-risk dataset patients of the International mRCC Database Consortium (IMDC), concluding no detectable difference in the OS among the therapies for these subsets of patients [33].

A specific subset of mRCC patients is represented by those with pancreatic or lung metastases only, often characterized by an overall good outcome. Indeed, RCC patients with pancreatic metastases showed a favorable prognosis compared to other sites of metastases [34, 35]. Interestingly, it has been observed that RCCs which metastasize to the pancreas are characterized by a higher sensitivity to antiangiogenic agents and resistance to ICIs [36•]. In a retrospective study involving 262 patients, the probability of survival after pancreatic metastases at 1, 3, and 5 years resulted in being 100%, 87.7%, and 78.9%, respectively. Compared to patients with non-pancreatic metastasis (median OS 2.7 years, *p* < 0.0001), the survival rate found in the study was significantly longer [37]. The lung is one of the most common metastatic sites in RCC [38], but their presence is anyway associated with a better outcome than metastases in other sites [39]. Interestingly, an analysis performed on pulmonary metastases characterized by spontaneous regression revealed 76% of RCC origin [40]. In addition, an observational study comparing OS in patients with pancreatic and lung metastases treated with TKIs alone or treated with surgical resection showed no significant differences between the two groups (OS: 86 vs 103 months respectively, *p* = 0.201) [41]. Given the same favorable outcome for single therapy as well as surgery for pulmonary metastases, combination therapy may be uncertain for patients with pancreatic or pulmonary disease.

There are other categories of patients for whom using a TKI alone might be advisable rather than a combination therapy [42–44]. A typical example is represented by patients with renal function impairment, usually not included in large prospective trials. The largest case history, including 39 RCC patients with renal function impairment treated with TKI (sunitinib or sorafenib), has been reported by the Cleveland group (USA) [45]. Although dose reduction was necessary for half of the patients due to increased creatinine levels, treatment efficacy remained substantially similar, both in terms of partial response and stable disease, for patients with preserved renal function [45].

It must be mentioned that immunotherapy is not suggested in the case of any autoimmune pathology and its administration must be carefully evaluated for patients in need of chronic immunosuppressant treatment. Therefore, ICIs also must be avoided in patients with autoimmune nephropathy [46]. Furthermore, the use of these agents should be carefully considered in those with a previous organ transplantation history in order to avoid organ rejection due to a strong immune response. Presently, few preclinical data are available in this regard combined with other case reports [47, 48•], with no clear indications from randomized controlled trials.

Elderly patients are also another category garnering a particular interest as half of the newly diagnosed renal cell carcinoma are found in patients > 65 years of age, predominantly 25% cases between 65 and 74, and another 25% cases in > 75 years old [49, 50]. There is a lack of significant data concerning the outcome of combination therapies in this population. Elderly patients in clinical trials are usually poorly represented due to several reasons: (1) a supposed greater risk of adverse events and therefore reduced tolerability of treatments and (2) the presence of comorbidities worsening performance status. Recent data report the comparison of ICI treatments (monotherapy or combination) in older patients with respect to young adults. No association has been found at multivariate analysis between older age and worst OS. However, older population displayed a shorter median OS compared to younger individuals (25.1 vs. 30.8 months) and lower ORR (24% vs. 31%, *p* = 0.01) likely due to a difference response in first-line treatment (31% vs. 44%, *p* = 0.02 for older and younger, respectively) [51•]. It is not possible to exclude a patient for a combination treatment based only on age [51•]. Nevertheless, elderly patients often present multiple comorbidities or difficulties accessing a treatment center for intravenous therapy on a periodic basis. Therefore, TKI alone could be considered a valid option for elderly patients.

Indeed, interesting results from the trials have shown the potential use of a single TKI as a therapeutic agent in this population.

In a recent study, around 32–40% of the patients (> 65 years old) enrolled with sunitinib [9, 52, 53] demonstrated comparable efficacy and adverse events to that of younger patients. A prospective observational study, PRINCIPAL, carried out in a real-world setting including 60% patients > 65 years (median age 66 years, range 22–90), confirmed the efficacy of pazopanib in terms of OS, PFS, and ORR [54].

Looking more closely at the age distribution of patients, it can be emphasized that a substantial improvement was observed in second-line PFS obtained with axitinib relative to sorafenib (6.7 vs. 4.7 months, HR 0.665; 95% CI 0.544–0.812; one-sided *p* < 0.0001) and a low percentage of discontinuation of therapy (4% vs 8%, respectively), in a population where 34% of the patients were 65 years or older [55]. Similarly, the METEOR study, which included 40% of patients > 65 years in second-line settings, showed amelioration in cabozantinib OS, PFS, and ORR compared to everolimus [56].

In light of the presented findings, it is questionable whether it is appropriate to expose specific subgroups (favorable prognosis patients, fragile patients, or subjects with multiple comorbidities) to combination therapy. In terms of saving of resources and toxicity, often preserving a good quality of life, the use of TKI monotherapy is still likely to be suggested for these subgroups of patients to achieve an overall good outcome.

On the other hand, some patients may present contraindications for TKI use. This happens in the case of predisposition to pathologies that are related to TKI-induced adverse events. In particular, hypertension is one of the most common adverse events associated with the use of TKIs, with an incidence of occurrence reported between 17% and 49.6% of patients [57]. Therefore, for those subjects already suffering from severe cardiovascular diseases, hypertension induced by TKIs could predispose to a higher risk of worsening of the condition and developing of cardiovascular events [57–59]. The molecular pathways involved in TKI-induced hypertension still need to be elucidated, and a genetic predisposition may be involved [57]. Nevertheless, the development of hypertension seems to be correlated with better TKI efficacy [60]. Specific attention should also be paid to patients at risk of hemorrhages as the TKI in association with anticoagulant therapy has been reported to increase the bleeding risk [61]. Similarly, fragile patients suffering from hepatopathies and elevated transaminase levels [62] or gastrointestinal problems [63, 64] may be exposed to a higher risk of exacerbations when treated with a TKI. In such conditions, therapy with an ICI as a first-line followed by a combination of two ICIs as a second-line may be a possible option, as recently explored with a multicentric European study (TITAN-RCC). This trial is designed to assess the outcome of treatment with nivolumab alone, followed by the addition of ipilimumab as a booster to improve the efficacy of the treatment and to reduce the rate of adverse events [65]. The association of PD-1 and CTLA-4 inhibitors may be better tolerated than TKI in some patients, but there are currently no factors predicting for immune-related toxicity to guide the clinical decision [66•].

## Immunomodulation by TKIs: Influence on Sequence and Combination Therapy

**Fig. 1:**
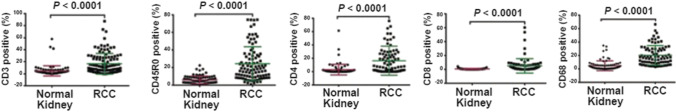
Immune cells in RCC, compared with normal kidney tissue. RCC, renal cell carcinoma. Modified from [67]

RCC is considered an immunogenic tumor, and a high number of immune cells are detectable within the tumor tissue, such as tumor-infiltrating lymphocytes (Fig. 1) [67, 68•]. Cancer development is delayed by mounting an effective immune response to the tumor [69]. Antiangiogenic agents have been shown to delay tumor progression not only by impairing angiogenesis in the tumor microenvironment but also by dampening the immune response of immunosuppressive cytokines and cells, like T reg cells [70, 71]. Since most TKIs have antiangiogenic capabilities, they can also boost the immune response. In this scenario, a TKI monotherapy can provide the required immune priming against the tumor, even without using ICI. Evidence of immune priming due to the modulation of angiogenetic molecules, in particular VEGF, has been provided by different studies, both directly (inhibition of the maturation of dendritic cells and proliferation of effector T cells, with up-regulation of PD-L1 expression, reduction of T reg cells) and indirectly (modulation of adhesion molecules and chemokine expression which in turn decreases the recruitment of immune cells within the tumor) [72, 73]. Bevacizumab and the combination bevacizumab–atezolizumab have been shown to reduce the level of inflammatory cytokines (IFNγ, IL-4, and IL-17), improve in vitro and in vivo cytotoxic T-lymphocyte response, and promote dendritic cell activation [74] and T-lymphocyte migration [75].

Furthermore, axitinib increases effective immune cell infiltration into the tumor tissue [76], cabozantinib actively downregulates suppressive myeloid cells and stimulates T-effector cells [77], and T regs appear to be decreased by sunitinib as well [78].

**Fig. 2:**
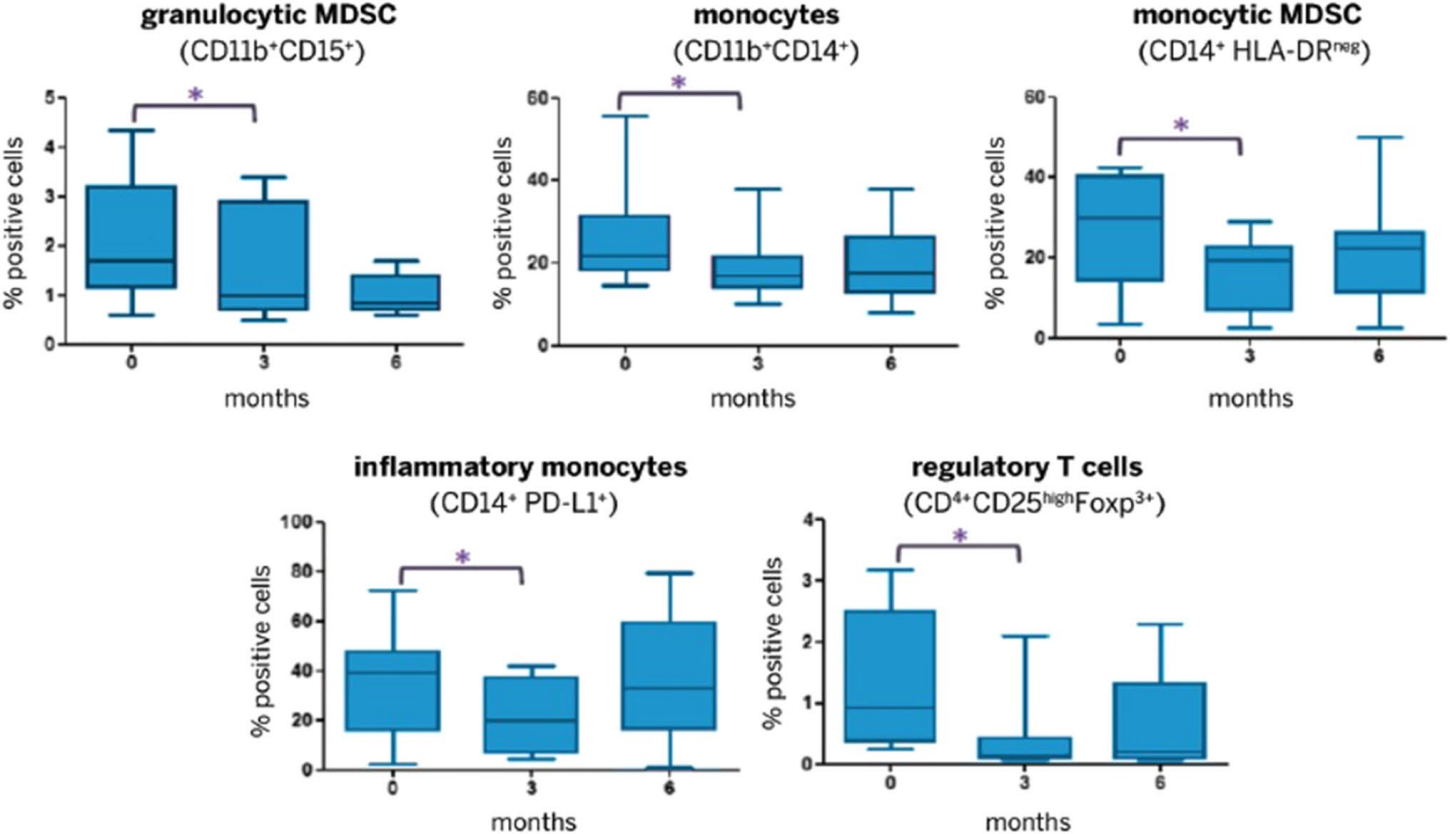
Reduction of immunosuppressive cells (MDSC and T reg) in peripheral blood during treatment with pazopanib for RCC. MDSC, myeloid-derived suppressor cells. Modified from [77]

Interestingly, pazopanib also appears to exhibit immunomodulatory actions. Verzoni et al. conducted a study on 16 patients treated with pazopanib as first-line for mRCC, analyzing blood immune profiling after 6 months of therapy. The drug showed a marked reduction in immune-suppressive myeloid cells, like T reg and myeloid-derived suppressor cells (Fig. 2).

These results were also supported by a study conducted in vitro on DCs from healthy donors [79••]. DCs from healthy subjects showed an increase of differentiation rate when exposed to pazopanib and exhibited greater T cells stimulation.

Data were confirmed by the clinical phase of the study, in which treatment with pazopanib significantly increased DC activation and a greater down-regulation of DC-regulated PD-L1 and IL-10 production [79••].

The immunomodulation activity can also be achieved through the reprogramming of the metabolism of the tumor cell. Indeed, RCC is characterized by a modification of several biochemical, cellular pathways. A switch toward increased glucose utilization (via glycolysis, glycogenolysis, pentose phosphate pathway) and an impairment of both oxidative phosphorylation and fatty acid metabolism, both mitochondrial pathways, is a signature of RCC cancer cells [80–83]. The metabolic signature of renal cancer cells is also characterized by increased use of amino acids such as glutamine and glutamate [80, 83]. As a consequence of this metabolic reprogramming, the tumor microenvironment displays a peculiar composition with several metabolic intermediates that can influence the response to treatment. The role played by metabolism in cancer therapies emerged in a study where, upon nivolumb treatment, a specific response ‒ increased serum kynurenine/tryptophan ratio – was associated with a worse outcome [84]. Moreover, the metabolic-modified tumor microenvironment may interfere with the behavior of the surrounding cells including immune cells. In fact, the administration of an adenosine receptor antagonist, a metabolic modulator, was associated with CD8 + T cells infiltration in tumor biopsies, underlying an interplay between metabolic pathways modulation and immune cell infiltration [68•]. The activation of specific metabolic pathways and the respective accumulation or depletion of intermediates can regulate angiogenesis and inflammatory features, thus influencing tumor fate [85]. The targeting of metabolic pathways is a field yet to be explored and may represent a new frontier in combination therapies with TKI, mTOR inhibitors, and ICI agents for the treatment of RCC [86].

In a sequential therapy rather than a combination, TKI’s ability to stimulate immune response could be an advantage. Some patients may benefit from immune priming first, initial tumor response, and specific immunotherapy subsequently. The scarcity of reports has made it difficult to assess which treatment option would be the best for triggering an immune response, whether TKI and ICI are used sequentially or TKI and immunotherapy are used simultaneously. To date, prospective data regarding the efficacy of different sequencing therapies are not available, as most of the studies are still ongoing and the data are therefore immature to draw any conclusion. One example is the Breakpoint Study, assessing the role of cabozantinib in patients pretreated with one ICI in monotherapy or in combination [87]. The evidence regarding the efficacy of targeted agents in patients pretreated with ICIs comes from retrospective analyses [88••, 89••].

ICIs can then synergize with antiangiogenic/TKIs [73] since there is a clear rationale for their use in the treatment of multiple tumors, including RCC [90].

Indeed, the expression of immune checkpoint receptors, particularly CTLA-4 and PD-1/PD-L1, represent pathways by which T-cell immunity is blocked [91]. Hence, they represent potential therapeutic targets, and their inhibition has led to a significant clinical benefit in the treatment of several types of tumors [73, 78, 92–94]. For mRCC patients, the combination of various immunotherapy agents seems to be a reliable alternative.

However, a variable proportion of patients (about 20–40%) are primary refractory to ICIs [95], and immunotherapy is useless for this subset of patients. In the absence of predictive factors that can identify these primary refractory patients, giving an extra drug could lead to a risk of additional toxicity without a clinically realistic benefit to the patient. In this case, the use of TKI monotherapy which could stimulate the immune response is a good alternative.

## Gut Microbiota Modulation in RCC

Gut microbiota has recently been included as a modulating immunotherapy factor that can influence the response to cancer therapy [96, 97•]. The microbiota plays a fundamental role in immune functions [98, 99]. Its alterations can cause anomalies in local and systemic immune responses, affecting cytokine secretion and T lymphocytes activation [97•, 100]. Specifically, alterations of the microbiota can influence the response and toxicity of antineoplastic therapies, as demonstrated by a large number of both preclinical [101, 102] and clinical [96, 103–106] data.

The impact of intestinal microbiota on the outcomes of treatment with ICIs is fascinating. A proportion of patients treated with ICIs have no, or not durable, responses. Emerging evidence suggests that alterations in the intestinal microbiota (often caused by previous therapies) can be included among the factors that affect immunotherapy resistance [104].

Gopalakrishnan et al. demonstrated that, relative to non-responders, patients responding to anti-PD-1 therapy for metastatic melanoma have a more differentiated microbiota [96].

Any alterations in the intestinal microbiome caused by previous therapies, such as TKIs, should be addressed to allow the restoration of physiological conditions. Ianiro et al. have reported the treatment of diarrhea caused by TKIs in RCC patients following fecal microbiota transplantation. It can be hypothesized that the results of subsequent therapies, particularly with ICIs, may be influenced by this manipulation of microbiota [107•]. Therefore, it is an important factor in defining personalized anticancer therapy, considering the wide range of individual variations, including differences in intestinal microbiota.

## Conclusions

The treatment and management of metastatic RCC patients have radically changed over the past 20 years [108••]. A great number of trials have demonstrated the efficacy of numerous molecules and combinations, prolonging the PFS and OS of RCC patients. An increased number of combination therapies, associating either an immune agent and TKI or two immune agents, represent new opportunities for RCC treatment and are beneficial for most patients. However, it is not excluded that some selected categories of patients may still benefit from monotherapy with TKI and a smart treatment sequence instead of a combination strategy. These patients may be a minority but still represented within the population: the ones with favorable risk and very long survival due to indolent disease, the ones with autoimmune pathologies, those normally excluded by clinical studies (patients with ECOG score > 1, patients with relevant cardiac or pulmonary comorbidities, renal impairment, elderly patients or patients with only pulmonary or pancreatic metastases), who are the real-world patients.

As the immunological response is essential in RCC, an effective immune stimulation could improve the patient outcome. Some TKIs have been shown to have immunomodulatory activity, and their use can prove beneficial in boosting the patient immune response against the tumor, even more, if preceding ICIs in the treatment sequence.

Currently, there is insufficient evidence to adopt the best strategy for promoting immune response, as some patients may benefit from a sequential therapy with a TKI followed by immunotherapy, while others may require a combination treatment TKI-immunotherapy upfront.

It is imperative to define the most appropriate treatment for each patient, and in some selected conditions, the use of TKIs can still represent a therapeutic option.

The available data, albeit limited, could provide a rationale for further studies aimed (e.g., by searching for specific markers) at identifying patients suitable for sequential TKI-immunotherapy.

## Data Availability

Not applicable.
